# Decreasing aerosols increase the European summer diurnal temperature range

**DOI:** 10.1038/s41612-025-00922-3

**Published:** 2025-02-12

**Authors:** Carla M. Roesch, Emilie Fons, Andrew P. Ballinger, Jakob Runge, Gabriele C. Hegerl

**Affiliations:** 1 School of GeoSciences, University of Edinburgh, Edinburgh, United Kingdom; 2 Institute of Atmospheric and Climate Science, ETH Zürich, Zürich, Switzerland; 3 Institute of Data Science, German Aerospace Center (DLR), Jena, Germany; 4 Technische Universität Berlin, Berlin, Germany; 5 Center for Scalable Data Analytics and Artificial Intelligence (ScaDS.AI) Dresden/Leipzig, Technische Universität Dresden, Dresden, Germany

**Keywords:** Attribution, Atmospheric science

## Abstract

The diurnal temperature range (DTR), the difference between daily maximum and minimum temperature, is important for the impact of extreme temperatures, but despite physical links to aerosol forcing previous studies have struggled to attribute observed DTR changes to aerosols. Using causal inference, we can clearly identify aerosols as a driver of European DTR change since 1940. Following a decrease from the 1940s, since the 1980s the European DTR has increased by about 0.5K due to a reduction in European aerosol emissions leading to cooler nights relative to days. Agreement between causal effects estimated from observations with those estimated for two CMIP6 models evaluates the models’ microphysical and radiative parameterizations. From causal effects, we also derive effective radiative forcing estimates of aerosols on surface shortwave during European summer, which amount to [−1.7; −1.5] Wm^−2^ in observations and one model, while it is less negative in the other model ([−0.9; −0.8] Wm^−2^).

## Introduction

The diurnal temperature range (DTR), defined as the difference between the daily maximum (${{\rm{T}}}_{\max }$) and minimum (${{\rm{T}}}_{\min }$) temperature, is an important measure of climate change and has impacts on both human (e.g., mortality^1,2^) and natural health (e.g., crop yields^3,4^, viticulture^5^, the ecosystem^6^). By reflecting changes in temperature extremes and the diurnal cycle, it can provide more information than daily mean temperature alone^7^.

**Fig. 1:**
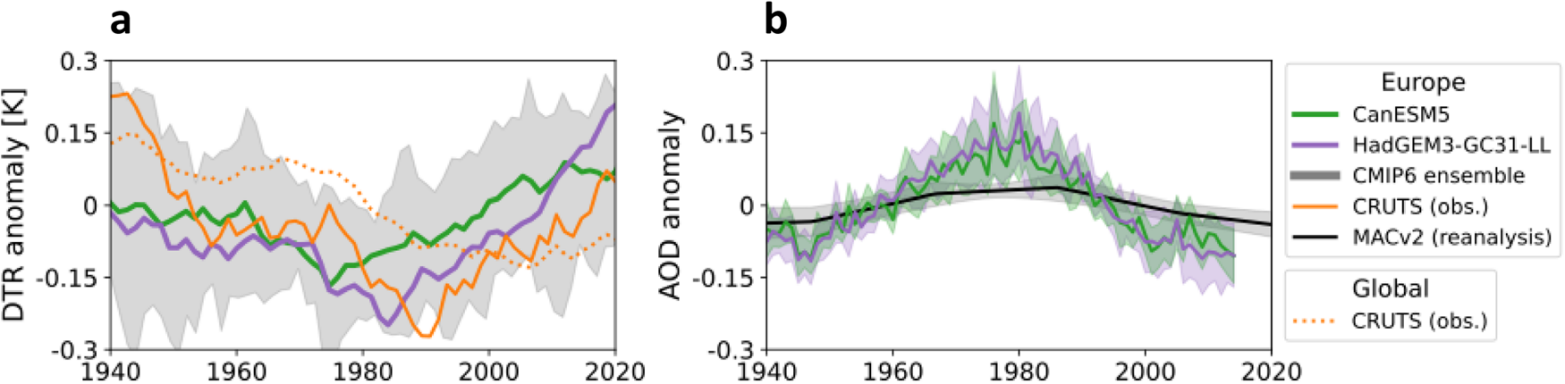
Observed and simulated anomalies of daily temperature range (DTR) and aerosol optical depth (AOD) for boreal summer (MJJA) relative to 1940-2020. AOD is a common measurement proxy for atmospheric aerosol concentrations^39^. Solid lines indicate a change in European average values, the dotted line indicates the global MJJA land mean. Observations for DTR and AOD are taken from CRUTSv4.06^79^ (orange) and MACv2^60^ (black), respectively. Model-ensemble average of CMIP6 historical ALL-forcing simulations^36^ from the CanESM5^58^ and HadGEM3-GC31-LL^74^ are also plotted in green and purple, respectively, with grey shading in **a** showing the combined 90% range of the CanESM5 (9) and HadGEM3-GC31-LL (4) ensemble. In **b**, due to the decadal resolution of MACv2 the time series appears more smoothed than for the two models.

By impacting Earth’s radiation budget through opposing radiative effects, human emissions of greenhouse gases (GHG) and aerosols (small liquid or solid particles that are suspended into the atmosphere) are suggested as dominant drivers of changes in the DTR^8,9^: GHG predominantly affect ${{\rm{T}}}_{\min }$ (usually observed in the early morning^8,10^) by increasing the absorption of outgoing longwave (LW) radiation, i.e., a reduction of planetary LW cooling at night^11,12^. GHG warming subsequently results in increased global mean temperatures but also a reduced DTR. Globally homogeneously distributed GHG concentrations are therefore assumed to drive the decreasing global mean DTR (dotted orange line in Fig. 1a)^12,13^. Aerosol concentrations, on the other hand, vary regionally^12,14^ and have a negative effective radiative forcing (ERF; [-2;-0.4] Wm^−2^^9^), offsetting some of the GHG-induced warming^15^. Aerosols either directly interact with incoming shortwave (SW or solar) and LW radiation through absorption or scattering (aerosol-radiation interactions, ARI), or indirectly by serving as cloud condensation nuclei (CCN) or ice-nucleating particles (INP) for cloud formation, modifying the radiative properties of clouds (aerosol-cloud interactions, ACI)^16^. Thus, aerosol effects on the DTR are manifold. For example, they can decrease ${{\rm{T}}}_{\max }$ through direct scattering of SW or increased cloud brightness^8,17^, but can also increase ${{\rm{T}}}_{\min }$ by trapping outgoing LW radiation through absorption and by increasing cloud cover at night^18,19^.

The complexity of these forcing impacts contributes to varying regional patterns^20,21^ such as a significant increase of the European DTR since the 1990s (solid orange line in Fig. 1a)^22,23^. A reduction in the European aerosol burden (Fig. 1b) has been proposed, but not confirmed, as a driver of this change by warming daytime and cooling nighttime temperatures (see Supplementary Fig. S1)^23,24^. Aerosol impacts on the DTR remain difficult to quantify and attribute in observations due to uncertainties in ACI^25^, high climate variability^14^ and uncertain DTR trends in global climate models^23,26,27^. These uncertainties have also led to large confidence intervals in aerosol ERF^28^.

Unlike previous studies that use model simulations to study DTR drivers^8,27^, in this study, we investigate aerosol effects on DTR changes in station and satellite observations using causal inference methods^29,30^. These methods have recently been applied in a number of climate-related studies to analyse ENSO teleconnections and mechanisms of the Walker circulation^31^, Arctic drivers of mid-latitude circulation^32^, the decoupling of photosynthesis and transpiration in Mediterranean woodlands^33^ or to study the impact of aerosol perturbations on stratocumulus clouds^34,35^. To disentangle aerosol effects on the DTR, we perform causal discovery and causal effect estimation on observations. Then, as a pilot study on using estimated causal effects for model evaluation, observational results are compared to causal effects from two climate models that are part of the 6th phase of the Coupled Model Intercomparison Project (CMIP6)^36^ and have sufficient data available^37,38^. Finally, estimated causal effects are used to reconstruct aerosol-induced DTR changes and to compute estimates of aerosol radiative forcing on surface shortwave for both observations and models.

## Results

### Causal discovery of aerosols effects on DTR

Before estimating the effects of aerosols on the DTR, we apply a causal discovery method^29^ to determine relationships between aerosols and potential mediators on the DTR. Since aerosols impact surface temperature through ACI and ARI^16^, aerosol optical depth (AOD; a common measurement proxy for atmospheric aerosol concentrations^39^), cloud area fraction (Clouds), incoming solar shortwave radiation at the surface (SW), ${{\rm{T}}}_{\max }$ and ${{\rm{T}}}_{\min }$ are considered as potential causal drivers of DTR. To discover the causal relations between these input variables (also called nodes), the causal discovery algorithm PCMCI+ (Peter Clark Momentary Conditional Independence)^40^ is used, which is implemented as part of the Python package tigramite(https://github.com/jakobrunge/tigramite). We focus our analysis on the boreal summer months (MJJA) because aerosol impacts on SW radiation in Europe peaks during these months^41^ and higher latitudes are exposed to little sunlight during winter. Further, we are expecting more stable cloud regimes during the summer months. We use MJJA daily station observations of cloud cover, ground-measured SW radiation, ${{\rm{T}}}_{\max }$ and ${{\rm{T}}}_{\min }$ from the European Climate Assessment & Dataset project (ECA&D)^42,43^ in the period 2001-2021. Sparse meteorological station data is used as ground truth during this causal discovery step because of inherent biases associated with satellite data, such as retrieval biases^39,44^ and spatial aggregation^45^. However, for AOD we have to rely on data at 550 nm from the gridded Clouds and Earth’s Radiant Energy Systems dataset (CERES; 1° x 1°)^46,47^, because there are no suitable in situ observations available (methods). ECA&D stations are not homogeneously distributed across Europe (Supplementary Fig. S2). Thus, to discover a mean European graph, individual years and stations are standardised and concatenated (considering time lags; see methods) in a single dataset (Supplementary Fig. A2). PCMCI+ is then applied to this multi-dataset and the causal links between the investigated variables are “learned” by making use of sequential conditional independence tests. Here, causal standard assumptions are made of time-order (cause precedes attributable effects), causal sufficiency (all common drivers in the system are observed), the Causal Markov condition (variables are independent from each other as stated by the causal graph), and faithfulness (observed conditional independencies arise from the causal structure)^29^. For the application of PCMCI+ we assume a significance level pc_alpha_ = 0.01 and a maximum time lag ${\tau }_{\max }$ = 1 (one day). As a first order approximation, we can assume linearity between the causal nodes (Supplementary Fig. S4), especially after annually standardizing the timeseries^33^. Thus, ParCorr, a linear independence test, is used to test for conditional independence.

When choosing the investigated causal drivers, a set of assumptions is made due to limitations in data availability. LW radiation is not explicitly included in the graph, but is expected to play a mediating role in the links of clouds and AOD on ${{\rm{T}}}_{\min }$ (Supplementary Fig. S5), which has no effect on the validity of our discovered graph. We also do not separate between different cloud types, e.g., high or low-level clouds, or aerosol species which can have different radiative forcing impacts. Estimated aerosol and cloud effects, therefore, represent net effects, and the discovered causal graph should be considered a net European mean. The temporal resolution of available model and observational data limits this study to daily values. However, aerosol effects can also take place on smaller time scales, and some effects, such as wet scavenging^48^, cannot be resolved (methods). We also acknowledge that a possible overestimation of AOD due to aerosol swelling and cloud contamination^39,44^ can result in the overestimation of the causal link from AOD to clouds due to confounding from relative humidity^49^. To prevent the discovery of spurious causal links, link assumptions based on our physical knowledge of the climate system are set (methods; Supplementary Table S1).

**Fig. 2:**
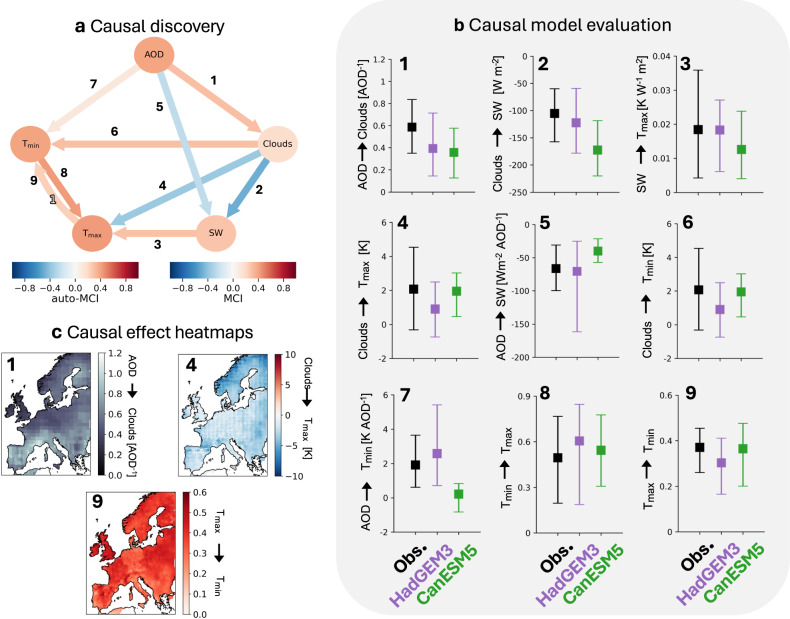
Causal analysis results. **a** Displays the discovered causal graph of aerosol effects on the DTR using PCMCI+ (significance level pc_alpha_ = 0.01, maximum lag ${\tau }_{\max }=1$, partial correlation test ParCorr, link assumptions as in graph C in Supplementary Table S1) on the standardised ECA& D multi-dataset. Nodes depict climate variables; arrows the direction of the causal links and curved arrows represent lagged links (outlined numbers, in days). Arrow colours depict the correlation sign and strength (MCI), node colour the autocorrelation (auto-MCI) of significant links. **b** Shows observed (Obs., black) and model (colour) non-standardised linear direct causal effect estimates (90% spatial variability) of mediated aerosol effects on DTR for the discovered graph in a. Effects are estimated using Wright’s method^53 – 55^ on individual gridboxes in the gridded E-OBS/CERES datasets. In **c**, heatmaps of causal effects in gridboxes in the E-OBS/CERES datasets are plotted (colourbars depict causal effect strength). Bold numbers (**1** to **9**) refer to climate effects in Table 1.

Figure 2 a shows the discovered causal graph where circles depict causal nodes, the direction of the arrows shows the direction of the causal effects, the colour of the arrows depicts the MCI partial correlation value, i.e., an indication of the sign and strength of the link, and curved arrows denote lagged links with the time lag marked as a outlined number. A sensitivity analysis of the discovered graph to varying link restrictions confirms that link assumptions are able to remove weak spurious links, while dominant links remain unchanged (compare graph A and C in Supplementary Fig. S6). Two additional sensitivity studies support our choice of season and variables. Causal discovery on the boreal warm period (April to October) reveals that the causal graph remains the same during the extended period (Supplementary Fig. S7). Additionally, using CAMS global reanalysis AOD^50^ of sulfate (SO_4_) and black carbon (BC) separately, we discover causal graphs for different aerosol species (Supplementary Fig. S8). We find the same causal signs but weaker effects for BC than SO_4_. Since we expect BC to warm the atmosphere through absorption and for sulfate to cool through scattering, these results further confirm the causal graph in Fig. 2a.

Confirming the validity of our discovered graph, we can compare aerosol effects from the literature to those estimated for each discovered link (columns 1 and 2 in Table 1). Our results, from observation, confirm that clouds, SW, ${{\rm{T}}}_{\max }$ and ${{\rm{T}}}_{\min }$ are climate drivers mediating the causal effect of aerosols (AOD) on the DTR: By serving as CCN/INPs, aerosols enhance cloud formation (link 1 in Fig. 2a; cloud fraction adjustment)^16^ which increases ${{\rm{T}}}_{\min }$ due to a reduction in outgoing LW (link 6). On the other hand, increased cloud cover decreases ${{\rm{T}}}_{\max }$ by both blocking incoming SW radiation (links 2,3) and through non-radiative effects, like evaporative cooling of precipitation (link 4)^51^. ARI (links 5,7)^16^ decrease ${{\rm{T}}}_{\max }$ but increase ${{\rm{T}}}_{\min }$ by reducing incoming SW during the day and outgoing LW at night. In summary, our discovered causal graph shows that while (increasing) aerosols raise daily minimum temperatures, they reduce daily maximum temperatures, thus, decreasing the DTR. Note that clouds as a causal node are represented by cloud area fraction in this study. Thus, changes to cloud albedo, e.g., Twomey effect^17^, are also included in link 5. Because ${{\rm{T}}}_{\min }$ occurs at night, no direct impact is expected from SW to ${{\rm{T}}}_{\min }$^39,52^.

**Table 1:** Discovered and estimated absolute (non-standardised) linear direct causal effects for the discovered graph (Fig. 2a)

Link	Climate effect	Causal sign	Causal effect estimate	Literature estimates
1	AOD → Cloud: aerosols serving as CNN/INPs and enhancing cloud formation (ACI, i.e, cloud fraction adjustment)^16^	+	0.6 [0.4; 0.8]	[0.59; 1.07] from^16^
2	Cloud → SW: increased cloud cover blocks SW from reaching the surface^9,80^	-	−105.3[−157.5; −59.9]Wm^−2^	[−177; 0]Wm^−2^ from^80^
3	SW $\to \,{{\rm{T}}}_{\max }$: surface warming by SW absorption^9,81^	+	0.02* [0.004; 0.04]	0.1K/Wm^−2^ from^81^
4	CC $\to \,{{\rm{T}}}_{\max }$: non-radiative cooling of clouds, e.g., evaporative cooling following precipitation^51^. This effect is only significant during summer and short time scales and the reversed, i.e, reduced precipitation, has been identified as a driver of summer heat waves^82^.	-	−3.3* [−6.05; −1.1]	−0.71^51^
5	AOD → SW: ARI^16^ through scattering and absorption. Because clouds are represented as cloud area fraction, cloud albedo changes (Twomey effect^17^) are also contained in this link.	-	−66.4*[−99.8; −30.95]	ARI: [−27; −20]Wm^−2^ from^16^ for global annual estimates
6	Clouds $\to \,{{\rm{T}}}_{\min }$: LW warming at night^9^	+	2.1 [−0.3; 4.5]	[1; 3] K^83^
7	AOD $\to \,{{\rm{T}}}_{\min }$ : LW warming through ARI at night^18,19^	+	1.9 [0.6; 3.6]	[0.39; 4.56] K (methods)
8	${{\rm{T}}}_{\min }\,\to \,{{\rm{T}}}_{\max }$	+	0.5 [0.2; 0.8];	[0;1] due to
9	${{\rm{T}}}_{\max }\,\to \,{{\rm{T}}}_{\min }$: temporal auto-dependency from sequential measurements	+	0.4 [0.3; 0.5]	temporal auto-dependency

Columns show in order: links (Fig. 2), associated climate effects, estimated causal signs, absolute direct causal effect estimates (European mean and spatial variability (90% range) from gridded E-OBS data) and estimates from the literature which are based on model and process studies. Link assumptions (see Graph C in Supplementary Table S1) are enforced during the discovery of the causal graph. Causal effect estimates marked with a (*) lie outside the range of literature estimates. Note, that literature estimates show global mean not European MJJA estimates.

### Causal model evaluation

**Fig. 3:**
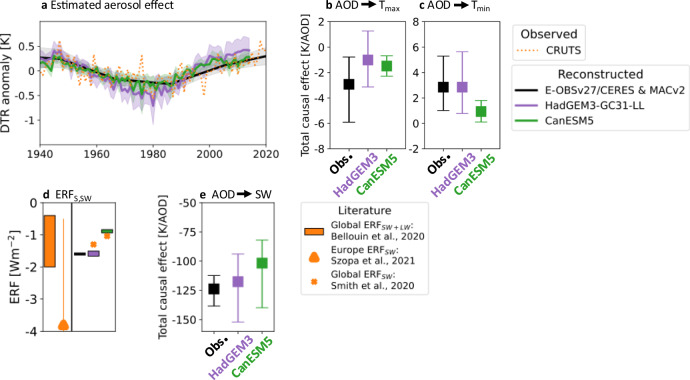
Estimated aerosol causal effects in models and observations. In **a** aerosol effects on DTR (1940-2014 for CMIP6 models in blue and purple; 1940-2020 for E-OBS and MAC; solid lines) are compared to observed (dotted orange line) DTR anomalies relative to 1940-2020. Total causal effects of aerosols on ${{\rm{T}}}_{\max }$ and ${{\rm{T}}}_{\min }$, which are used to estimate aerosol effects on the DTR, are shown in **b** and **c**, respectively. In **d** the estimated surface contribution to ERF_SW_, referred to as ERF_S,SW_, is compared between observations (black) and two CMIP6 models (purple and green). ERF estimates are obtained by multiplying the difference in AOD between 1850 and 2014 in models/[2005-2015] in observations with the total causal effect (TCE; in **e**) of aerosols on SW. For comparison, global annual aerosol ERF^16^ (orange bar), multimodel mean European aerosol ERF_SW_^28^ (orange triangle) and aerosol ERF_SW_ for CanESM5 and HadGEM3-GC31-LL^19^ (orange crosses) are plotted in **d**.

Results from the PCMCI+ algorithm only give an indication of the sign and strength of the discovered causal link (methods). To further validate the causal graph and to compare our findings with results from the literature, the strength of detected causal effects from observations is estimated using Wright’s method^methods; 53–55^. ${\alpha }_{{X}^{j},{X}^{i},\tau }$ describes the linear direct causal effect of a discovered causal link from *X*^*j*^ on *X*^*i*^ at lag *τ* and essentially represents the regression coefficient of *X*^*j*^ on *X*^*i*^ and hence allows to quantify the response to aerosols in observations similar to done in fingerprint detection and attribution methods^15^. Different to a regular linear regression, potential confounding factors are removed prior based on the input graph. For a spatial analysis, we retrieve data for ${{\rm{T}}}_{\min }$, ${{\rm{T}}}_{\max }$ and SW from the high resolution E-OBSv27.0e gridded dataset (0.25°x0.25°)^56^. Cloud area fraction and AOD are retrieved from the CERES dataset. Because we will compare these results with model estimates we limit the analysis to the period 2001-2014 due to the availability of CERES (2001-2023) and historical CMIP6 simulations (1850-2014). Then, absolute (i.e., non-standardised) linear direct causal effects are calculated for each grid box for the discovered causal graph (methods). The European mean and 90% spatial variability of the resulting causal effects are plotted in black in Fig. 3b. Causal effects derived from E-OBS agree with those from ECA&D station data (Supplementary Fig. S9).

There is a spread in causal effect strengths across Europe due to local effects such as the spatial heterogeneity of aerosol emissions and composition (links 1, 5, 7), cloud types (links 1, 2, 4, 6) or surface cover type (links 4, 6, 8, 9). Heatmaps of the estimated causal effects (Fig. 2c and Supplementary Fig. S9) highlight this spatial variability (e.g., elevation effects in link 4 in Fig. 2c) but also reveal artefacts in the underlying gridded data, such as grid lines^45^, which is especially true for AOD (see kink 1 in Fig. 2c), and country borders (e.g., Germany in link 9 in Fig. 2c). Still, causal effect estimates agree with sensitivity studies from the literature as shown in Table 1.

Following the same approach as for the gridded observations, we estimate absolute linear direct causal effects for every grid box in historical CMIP6^36^ simulations for HadGEM3-GC31-LL (1.88° × 1.25°)^57^ and CanESM5 (2.8° × 2. 8°)^58^ using data for the period 2001-2014 (sufficient data for the causal analysis were only available for these models at the time of writing, see methods). Both models have prognostic aerosol schemes and simulate ARI and ACI (Twomey effect^17^, cloud lifetime and cloud liquid water adjustment due to precipitation suppression^16^)^57,59^. Evaluating the (micro-)physical parameterizations of these two models, estimated effects for the models agree with those from observations although the spatial spread varies. Potentially explaining stronger trends in DTR changes (Fig. 1a), we find higher effects from aerosols (links 1, 5 and 7 in Fig. 2b) in HadGEM3-GC31-LL than in CanESM5. This also aligns with recent studies on aerosol forcing in CMIP6 models, where HadGEM3-GC31-LL is found to have an aerosol ERF on SW of -1.30 Wm^−2^ while CanESM5 is estimated to have -1.04 Wm^−2^^19^. Due to the coarser resolution of CanESM5, spatial variability for this model is more constrained across Europe than for HadGEM3-GC31-LL.

### Estimated aerosol effect on DTR

Although a standard assumption in causal inference is that causal effects are stationary, i.e., causal graph and effects are constant in time, changes in AOD (Fig. 1b) can lead to variations in the time series of the DTR. From estimated direct causal effects we reconstruct the aerosol effect on the European MJJA DTR (Fig. 3a; relative to 1940-2014 in models and 1940-2020 in observations) by determining the total causal effects of aerosols on ${{\rm{T}}}_{\max }$ and ${{\rm{T}}}_{\min }$ (Fig. 3b and c) using the estimated causal relationships from day-to-day variability. These are given as the sum of the product of all path coefficients (i.e., direct causal effects) along all paths from AOD to ${{\rm{T}}}_{\max }$ and ${{\rm{T}}}_{\min }$ (Eq. (1) in methods). Finally, historical aerosol-induced changes in DTR are estimated by multiplying the difference of the total causal effects with the respective MJJA AOD anomalies relative to 1940-2014 (models) and 1940-2020 (observations). Reanalysis AOD data from MACv2^60^ going back to 1850 are used for observation-like estimates (methods).

Daytime warming and nighttime cooling, due to decreasing aerosol emissions, can be seen in the estimated total causal effect of AOD on ${{\rm{T}}}_{\max }$ and ${{\rm{T}}}_{\min }$, resulting in a European MJJA DTR increase since the 1980s (see Supplementary Fig. S10). Figure 3 shows that aerosol forced DTR anomalies estimated using causal effects explain the long-term evolution of DTR, confirming that aerosols are the dominant cause of DTR variability, and supporting our causal graph results. Even though models show weaker aerosol effects (especially CanESM5), this is compensated by higher AOD anomalies (Fig. 1b), which results in similar reconstructed contributions of aerosols to changes in DTR from CanESM5 and observations of about 0.5K since the 1980s. A stronger change in DTR is reconstructed for HadGEM3 of nearly 1K in the past 40 years. This is due to a higher estimated aerosol effect on ${{\rm{T}}}_{\min }$ (see Supplementary Fig. S10) of about 0.7K, while observations and CanESM5 only show an aerosol-induced change of about 0.3K. Aerosol impacts on ${{\rm{T}}}_{\max }$ are similar for both models and observations (about 0.4K). The lowest DTR was found around 1980 for both models and observations. Finally, the good agreement between the upscaled aerosol causal effects from this study and observed DTR changes (in orange) suggests that the important mechanisms for long-term DTR variability have been captured by the causal analysis.

### Effective radiative forcing estimation

From the total effect of AOD on SW (Fig. 3e), we can derive the total aerosol-induced change of SW radiation reaching the ground (links 1, 2 and 5). This allows us to quantify the surface contribution to aerosol ERF on SW, ERF_S,SW_ (Fig. 3d), defined as the difference in the surface component of reflected SW at the top-of-the-atmosphere (TOA) between pre-industrial (1850) and present day (Fig. 5). Note, due to conventions in the literature, “present day" is defined as 2014 for models and the mean of [2005-2015] for observations. We also assume that the European surface albedo has remained unchanged since 1850, which is a good approximation for the summer months^61,62^.

Generally, observational studies do not separate aerosol ERF in LW and SW components, in regional or seasonal estimates (global-annual: [−2;−0.4] Wm^−2^^16^). However, to put our results in perspective model studies have found that aerosol ERF is stronger in Europe than globally and the best estimate for European aerosol ERF_SW_ is around -3.8 Wm^−2^ with a spread of [−7; −0.5] Wm^−2^ in models^28^ (orange line and triangle in Fig. 3d). However, this estimate represents the full annual aerosol ERF_SW_. This means that it also includes the ERF contribution of SW radiation reflected from clouds and aerosols (Fig. 5), which we cannot quantify but contributes about 60% of the total TOA reflected SW radiation^63^. This supports both our observational estimates for European summer ERF_S,SW_ of [−1.6; −1.5] Wm^−2^ and modelled estimates of [−1.7; −1.5] Wm^−2^ for HadGEM3 and [−0.9; −0.8] Wm^−2^ for CanESM5. Findings for CanESM5 and HadGEM3-GC31-LL slightly deviate from estimates of global annual aerosols ERF_SW_ of −1.04 Wm^−2^ and −1.3 Wm^−2^ respectively (orange crosses in Fig. 3d)^19^. Note, that ERF_S,SW_ estimates from this study are better constrained than findings from the literature because only statistical and no systematic uncertainties can be considered (methods).

## Discussion

In this study, a causal graph of the effect of aerosols on ${{\rm{T}}}_{\min }$ and ${{\rm{T}}}_{\max }$ is discovered from station and satellite data and aerosols are identified as a significant driver of the European summer DTR, something previous studies have struggled to achieve^14,23,27,64^. Confirming its robustness, our discovered causal graph is consistent with physical expert knowledge of aerosol effects on the climate system (Table 1): the effects of ACI and ARI can be resolved and show that a reduction in European aerosol emissions has led to warming of daytime and cooling of nighttime temperatures since the 1980s, i.e., increasing the DTR by about 0.5K.

From the estimated total causal effect of aerosols on SW, we can further reconstruct the aerosol ERF on surface SW in Europe ([−1.6; −1.5] Wm^−2^). To our knowledge, this is the first time that the aerosol ERF of surface SW is estimated from observations. Changes in surface SW radiation have significant and far-reaching impacts not only on surface temperature (i.e., regional meteorology) but also on solar power generation^65^. Results from this study agree well with recent model estimates of European aerosol ERF_SW_^28^.

In a pilot study for climate model evaluation of HadGEM3-GC31-LL and CanESM5, we use the observations discovered causal graph and find that causal effects for aerosols and clouds are generally weaker in models than in observations. For the DTR, this is compensated by a higher historical AOD in the climate models, leading to similar reconstructed aerosol impacts. However, model ERF_S,SW_ estimates deviate between the models with HadGEM3 agreeing with the observations (due to higher model AOD but weaker causal effects) while CanESM5 shows a weaker ERF_S,SW_ ([−0.9; −0.8] Wm^−2^). Based on our study this suggests an underestimation of the aerosol forcing in the two models.

By estimating the aerosol effect on the DTR and their ERF from observations, this study serves as a pilot study highlighting the potential of causal inference for applications in climate sciences. Not only do these methods allow for observational sensitivity studies to external climate drivers, but through the direct comparison of causal effects from models and observations, there is also the potential to reduce model uncertainties through observational constraints.

## Methods

**Fig. 4:**
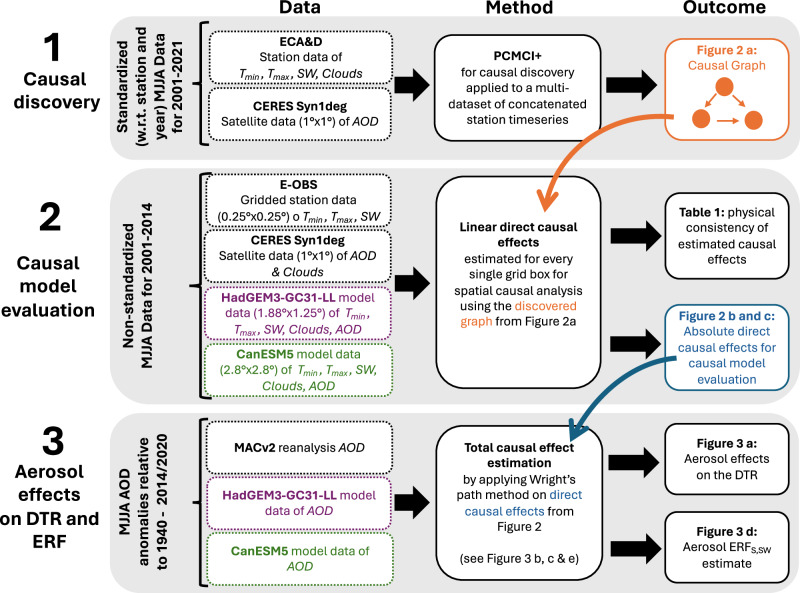
Schematic of our causal analysis workflow. The figure shows the required data and the applied methodology for each individual analysis step. Coloured arrows further highlight how results from previous steps propagate through the analysis. Rows depict individual analysis steps in order of the paper. Columns highlight first the required data, then the methodology and lastly the outcome of each step.

For the causal analysis in this study, we use the freely available Python package tigramite (https://github.com/jakobrunge/tigramite). Our analysis consists of three steps as described in the result section of this paper and in Fig. 4. The individual analysis steps further serve as subsections for this method section, where the used data and methodology is described in detail.

### Causal discovery

Causal discovery is applied to discover the causal relationships between aerosols and the DTR. Here, we use ECA&D daily station data^42,43^ for daily minimum and maximum temperature, cloud cover and incoming SW radiation as it has not been regridded. We only consider stations for which at least 10 years of European MJJA (May-August) observations are available between 2001 and 2021. This leaves n = 503 stations distributed across Europe (12W-40E, 20N-80N) (Supplementary Fig. S2). Because no suitable in-situ observations are available for aerosols, gridded aerosol optical depth (AOD) from CERES (1°x1°)^46,47^ is used as aerosol observations and individual stations are matched by finding the CERES gridbox with minimum distance for each station location (based on latitude and longitude). We initially considered using AERONET data^66^. However, they provide only very local information and even though the station coverage is relatively good in Europe it did not provide a comprehensive cover. Studies have found only a minimal negative bias in CERES data relative to AERONET station data in Europe^67^. To obtain a mean causal graph representative of the ECA&D dataset, causal discovery is conducted on a “multi-dataset", where all years and ECA&D stations are concatenated in a single MJJA time series for each variable. Because potential confounding arises from combining different stations (e.g., through elevation or latitudinal effects), we standardize the data for each year and station by subtracting yearly MJJA means and divide the anomalies by the yearly MJJA standard deviation.

Causal relationships between the selected variables are discovered by applying the PCMCI+ (Peter Clark Momentary Conditional Independence) algorithm to a time series dataset. The algorithm is included in tigramite and is built on the PCMCI algorithm thus allowing for the discovery of not only lagged (time lag *τ* > 0) but also contemporaneous (*τ* = 0) causal links. A detailed description of the framework and an analysis of the challenges of applying the algorithm to time series data can be found in refs. ^38,40,68^.

The PCMCI+ algorithm aims to construct the causal graph of an underlying discrete-time structural causal system *X*_*t*_ of *N* variables (also referred to as nodes) ${X}_{t}=({X}_{t}^{1},...,{X}_{t}^{N})$ in three phases. In the first two phases, starting from a fully connected causal graph, causal links are iteratively removed when found independent based on (momentary) conditional independence testing and lagged links are time-ordered. In the final phase a set of rules is applied (rules R1-R3^40^) to guarantee that no cycles occur. The result is a causal graph for which we assume the following standard assumptions: *common cause principle*, which implies that an observed dependency between variables is either due to them being causal to each other or that they are driven by a common driver (see^68,69^), *time order*, meaning that a cause precedes its attributable effects, *causal sufficiency*, namely that all direct common drivers in the system are observed, *causal Markov condition* which implies that in a causal graphical model a variable is independent of every other variable that is not affected by it given its direct causes and *faithfulness* which states that all observed conditional independencies arise from the causal structure.

The discovered causal graph can contain directed (" ⟶ ") lagged (*τ* > 0) and contemporaneous causal links (*τ* = 0), and unorientated contemporaneous links. Unorientated contemporaneous links are found when adjacency indicates Markov equivalence ("o—o") or the direction cannot be identified due to conflicting orientation rules ("x—x"), see Supplementary Fig. S6 for examples.

We set the parameters for the application of PCMCI+ (as implemented in tigramite) as the following: the significance level (*α*_*PC*_) for conditional independence testing is set to *α*_*PC*_ = 0.01, the maximum time lag (*τ*_*max*_) is assumed to be *τ*_*max*_ = 1 (one day, the investigated effects are known to work on short time scales), and the minimum time lag (*τ*_*min*_) is set to be *τ*_*min*_ = 0 to investigate contemporaneous links. To test conditional independence we apply partial correlations (*ParrCorr()*).

PCMCI+ also allows for the selection of links that should be “blocked”, referred to as link assumptions. These are set based on our expert understanding of the underlying structural causal system, i.e., the physical climate system. Thus, to reduce uncertainty and the potential of spurious links during the causal discovery, the following link assumptions are made:

1. ${{\rm{T}}}_{\min }$ is generally measured in the early morning before sunrise, thus it can be assumed that there is no (direct) link from SW radiation^52^.
2. On short time scales, cloud formation can be impacted by surface ${{\rm{T}}}_{\min }$ and ${{\rm{T}}}_{\max }$ through the evaporation of soil moisture and relative humidity. However, these effects are especially important for low-level and convective clouds, and appear on sub-daily time scales, thus are not resolved within this causal framework^70^. Thus, no possible feedback loops from ${{\rm{T}}}_{\min }$ or ${{\rm{T}}}_{\max }$ are considered in this study and AOD represents the only potential parent of clouds (out of the variables included in this study).
3. It is assumed that aerosol impacts on ${{\rm{T}}}_{\max }$ are mediated either through the interaction with clouds (ACI) or the direct interaction with incoming SW radiation (ARI). Hence, direct causal links from AOD to ${{\rm{T}}}_{\max }$ are blocked.

### Causal effect estimation

Discovered link strengths from PCMCI+ indicate the Momentary Conditional Independence (MCI) partial correlation values, and only give an indication of the orientation and sign of the causal link. Thus, to obtain causal effect strength, Wright’s method of path coefficients^53–55^ is applied in a second step. This allows for both the physical validation with literature estimates of the discovered causal graph but also for causal model evaluation. Wright’s method only applies in the linear case (assumed here) and allows for the estimation of the direct causal effects ${\alpha }_{{X}^{j},{X}^{i},\tau }$, which are obtained as the regression coefficients of a multivariate linear regression of ${X}_{t}^{j}$ on its parents (direct causes), as previously discovered through PCMCI+. Confidence intervals for the estimated direct causal effects are derived by applying a bootstrapping method with 1000 members and significance is assumed if the resulting 90% range does not include 0.

Due to data limitations, there are some effects that cannot be resolved in our study. Their potential impacts on our results are the following:

1. Due to large uncertainties in the required data set and limited observations, LW radiation is not explicitly included in the graph. It serves, however, as a mediator for the aerosol and cloud impacts on ${{\rm{T}}}_{\min }$ (purple links in Supplementary Fig. S5). This has no effect on the discovered causal graph or the estimated causal effects as LW does not represent a confounder, i.e. does not causally drive more than 1 variable in our graph (Supplementary Fig. S5).
2. Different cloud types and aerosol species vary in their radiative impacts, possibly resulting in opposing causal effects^16^. However, early attempts to causally distinguish between them have resulted in large uncertainties and physical inconsistencies due to data availability and complex feedbacks^71^. Thus, the discovered causal graph and estimated effects should be considered to represent the “net” interaction between aerosols and clouds (see Supplementary Fig. S8).
3. The temporal resolution of the available model and observational data limits this study to daily values, thus shorter-term effects, such as wet scavenging (removal of aerosols from the atmosphere through precipitation, which can happen on time scales of 0.3 to 1.5 days for stratiform clouds^72^), cannot be resolved. This means that clouds can impact AOD by reducing aerosol concentrations at a time lag of 1, but this link is not directly discovered by PCMCI+. Although this could potentially lead to an overestimation of the direct causal effect from AOD on clouds, it would not change the sign of the link.
4. The possible overestimation of AOD due to cloud contamination (i.e., stray cloud particles, particle swelling by humidification, shadows, and enhanced scattering into the aerosol field from clouds)^44^ cannot be fully corrected for in this study. We, therefore, acknowledge that the causal link from AOD to clouds can be overestimated in magnitude (not in sign), i.e., confounded by relative humidity in the immediate proximity of cloud formation.

Note that other variables such as GHG or vegetation, which might impact European DTR are not explicitly included in the causal analysis, as they represent external drivers (i.e., they are not confounders)^73^. Relevant to this study are variables that mediate the causal effect of aerosols on the DTR.

Because ECA&D stations are not homogeneously distributed across Europe, gridded ${{\rm{T}}}_{\min }$, ${{\rm{T}}}_{\max }$ and SW at the surface from the E-OBS dataset (0.25°x0.25°)^56^ are obtained to test the spatial robustness of the estimated causal links (see Figure S9 for comparison). Gridded observations of clouds and AOD are taken from CERES (1°x1°)^46,47^. To avoid introducing averaging biases, the datasets are not regridded but are matched by finding the CERES gridbox with minimum distance for each E-OBS grid box (based on latitude and longitude)^34,45^.

### Causal model evaluation

As a pilot study, we reproduce the observational study for causal model evaluation using model simulations at a daily resolution. Out of the CMIP6^36^ cohort, daily historical simulations of minimum and maximum temperatures, cloud area fraction, surface downwelling shortwave flux in air, and AOD at 550nm are only available for CanESM5^58^, HadGEM3-GC31-LL^74^ and NorESM2-LM^75^ (at the time of writing this paper; April 2024). However, due to inconsistencies in NorESM2 daily AODs (see https://github.com/NorESMhub/noresm2cmor/issues/324), model simulations are downloaded for CanESM5 (r1i1p1f1) and HadGEM3-GC31-LL (r1i1p1f3) only. Our analysis is limited to only one ensemble member, because only a single AOD simulation with a daily resolution is available for either model. As for the CERES and E-OBS datasets, the model simulations are not regridded to maintain the initial model causation.

Causal effects are then estimated for the discovered causal graph in Fig. 2a on individual grid boxes in the E-OBS and model data, where all years (MJJA) are concatenated in single time series for each grid box. Potential confounding from slow effects (e.g., global mean temperatures) work on different time scales to what is considered in this study (contemporaneous and daily). Therefore, absolute effects for individual grid boxes can be estimated from the non-standardised time series data. A comparison between absolute (non-standardised) effects for E-OBS and ECA&D shows that the ECA&D-derived European mean (black line in Supplementary Fig. S9) lies in the center of the E-OBS distribution (blue histogram in Supplementary Fig. S9). The only exception is link 4, due to the different units of the cloud datasets, which is cloud area fraction in E-OBS and Okta/8 in the ECA&D station data.

### Aerosol causal effects on DTR and ERF

From the discovered causal graph and estimated direct linear causal effects, the total causal effect *β*_*X*,*Y*,*τ*_ of *X* on *Y* can be calculated using Wright’s path tracing formula^53–55^:

1 $${\beta }_{X,Y,\tau }=\sum _{\begin{array}{c}\,{\rm{causal}}\\ {\rm{paths}}\, {\rm{from}}\\ X\,(t-\tau )\,{\rm{to}}\,Y\,(t)\end{array}}\prod _{\begin{array}{c}{\text{link}}\\ {X}^{j}\to {X}^{i}\\ {\rm{in}}\, {\rm{path}}\\ {\rm{at}}\, {\rm{lag}}\,\tau \end{array}}{\alpha }_{{X}^{j},{X}^{i},\tau }$$

As for the direct causal effects, confidence intervals for the total causal effects are derived by applying a bootstrapping method with 1000 members and significance is assumed if the resulting 90% range does not include 0.

Using the total causal effects *β*_AOD,Tmin_ and *β*_AOD,Tmax_, **aerosol effects on the European DTR** can be estimated from changes in the atmospheric aerosol loading (ΔAOD):

2 $$\begin{array}{r}{\text{DTR}}_{{\rm{anom}}}=({\beta }_{{\rm{AOD,Tmax}}}-{\beta }_{{\rm{AOD,Tmin}}})\cdot \Delta \text{AOD}\,\end{array}$$

Since models only cover the period until 2014, ΔAOD is estimated as anomalies relative to [1940;2014] in models and [1940;2020] in observations. AOD anomalies are derived from the respective model simulations. Since CERES starts only in 2001, historical AODs are reconstructed from monthly reanalysis AOD maps of the Max Planck Aerosol Climatology version 2 (MACv2;^60^), which are available in steps of 20 years from 1850-2100. We use linear interpolation to obtain annual AOD estimates for the years where no aerosol product is available.

**Effective radiative forcing** (ERF) has been introduced^16,76^ to quantify both the instantaneous radiative forcing (e.g., Twomey effect^17^) and the radiative forcing of rapid adjustments *R*_*atm*_, such as cloud fraction adjustments, to Earth’s radiative budget. Earth’s radiative budget is represented by the net radiative flux R at the top of the atmosphere (TOA), which in an equilibrium state remains constant because incoming and outgoing radiation balance each other. Thus, R is defined as:

3 $$R={R}_{\,\text{SW}\,}^{\downarrow }\cdot (1-a)-{R}_{LW}^{\uparrow }\approx 0$$

where ${R}_{\,\text{SW}\,}^{\downarrow }$ describes the incoming (solar) SW radiation at TOA, ${R}_{\,\text{LW}\,}^{\uparrow }$ outgoing (terrestrial) LW radiation at TOA and *a* the albedo, scaling the fraction of incoming SW radiation that is scattered back to space. We do not assume *R* ≈ 0, as perturbations to the radiative balance, due to natural (e.g., astronomical parameters, volcanic eruptions) or human causes (e.g., aerosols), result in an imbalance of Earth’s energy budget^16^. The system responds to these radiative changes by eventually reaching a new equilibrium state. The ERF of a given climate forcer (e.g., aerosols) is given by:

4 $$\begin{array}{r}\,\text{ERF}\,=\Delta R+\Delta {R}_{atm}\\ =\Delta ({R}_{\,\text{SW}\,}^{\downarrow }\cdot (1-a))-\Delta {R}_{LW}^{\uparrow }+\Delta {R}_{atm}\end{array}$$

Since we do not explicitly resolve causal effects on LW, we only estimate the ERF of aerosols on SW, which can be estimated as the difference of the net TOA SW fluxes between 1850 and present-day due to variations in AOD:

5 $$\begin{array}{r}{\text{ERF}}_{{\rm{SW}}}={({R}_{{\rm{SW}}}^{\downarrow }-{R}_{{\rm{SW}}}^{\uparrow })}_{{\rm{TOA}}}^{{\rm{1850}}}-{({R}_{{\rm{SW}}}^{\downarrow }-{R}_{{\rm{SW}}}^{\uparrow })}_{{\rm{TOA}}}^{\text{present day}\,}\end{array}$$

because ${R}_{\,\text{TOA,SW}\,}^{\downarrow }$ is not forced by aerosols, i.e., constant, eq. (5) is reduced to:

6 $$\begin{array}{r}{\text{ERF}}_{{\rm{SW}}}={R}_{{\rm{TOA,SW,present}}\,{\rm{day}}}^{\uparrow }-{R}_{{\rm{TOA,SW,1850}}}^{\uparrow }=\Delta {R}_{\text{TOA,SW}\,}^{\uparrow }\end{array}$$

**Fig. 5:**
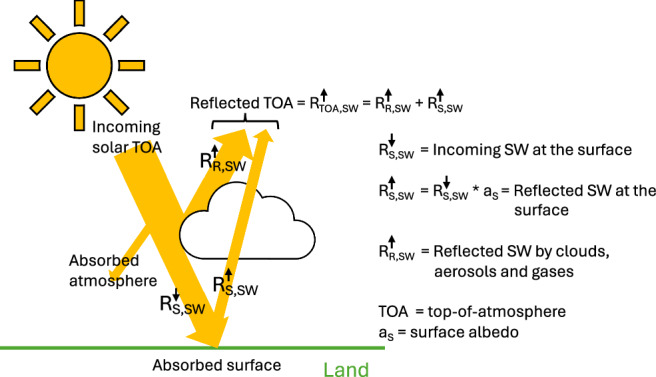
Schematic of the SW energy balance of Earth over land. Arrows show the fluxes discussed in this section to estimate contributions to aerosols ERF. Compare ref. ^84^ for estimates of global mean fluxes.

As can be seen in Fig. 5, ${R}_{\,\text{TOA,SW}\,}^{\uparrow }$ is a combination of incoming SW that is reflected by clouds, aerosols and gases (${R}_{\,\text{atm,SW}\,}^{\uparrow }$) as well as solar radiation reflected from the surface (${R}_{\,\text{S,SW}\,}^{\uparrow }$) with

7 $$\begin{array}{r}{R}_{\,\text{S,SW}}^{\uparrow }={R}_{\text{S,SW}\,}^{\downarrow }* {a}_{s}\end{array}$$

where *a*_*s*_ is the surface albedo.

Because both changes in cloud albedo (Twomey effect^17^) and ARI are included in *α*_AOD,SW,0_, i.e., the direct link from AOD to surface SW, we cannot calculate the contribution of SW reflected from aerosols or clouds and only estimate the contribution of reflected SW from the surface ERF_S,SW_ through the total effect of AOD on SW:

8 $$\begin{array}{ll}{\rm{ERF}}_{{\rm{S}},\,{\rm{SW}},\,{\rm{AER}}}={a}_{s}* {\beta }_{AOD,\,SW}* \Delta {\rm{AOD}}\,\\ \qquad\qquad\qquad\,=0.2* {\beta }_{AOD,\,SW}* \Delta \,{\rm{AOD}}\,\end{array}$$

where *a*_*s*_ is estimated to be 0.2 on land in Europe during the summer^77^. ΔAOD is derived differently for observations and models, because ERF in CMIP6 models is calculated as 2014 relative to 1850^19^ and in observations as [2005-2015] mean relative to 1850^16^.

ERF estimates are a net average measurement, thus, we do not consider spatial variability in our uncertainty estimation and derive confidence intervals for European mean causal effects using bootstrapping. The final ERF uncertainties are then estimated following:

9 $$\begin{array}{r}\Delta {\text{ERF}}_{{\rm{S,SW,AER}}}={\text{ERF}}_{{\rm{S,SW}}}* 0.2* \sqrt{{\left(\frac{\Delta (\Delta \text{AOD})}{\Delta \text{AOD}}\right)}^{2}+{\left(\frac{\Delta {\beta }_{{\rm{AOD,SW}}}}{{\beta }_{{\rm{AOD,SW}}}}\right)}^{2}}\end{array}$$

We derive Δ(ΔAOD) in the observations as the 90% temporal variability divided by 10 (number of years) as we are taking 10-year means. Uncertainties in ΔAOD are therefore not considered for model estimates. Uncertainties in surface albedo are not considered as MJJA surface albedo in Europe has remained approximately constant^61,62^. Since the causal effects are not normally distributed, we estimated ERF uncertainties for positive and negative deviations from the best estimate individually, to maintain the skewness using 90% confidence ranges for ΔAOD and *β*_AOD,SW_.

### Comparison of causal effect of AOD on ${{\rm{T}}}_{\min }$

In the following, we outline our approach to validate the causal effect estimates of AOD $\to \,{{\rm{T}}}_{\min }$ (Table 1) using values from the literature. Note, that this is just an approximation of the causal effect. The LW-mediated AOD causal effect is given through:

10 $$\begin{array}{r}{\alpha }_{{\rm{AOD}},{{\rm{T}}}_{\min },0}={\alpha }_{{\rm{AOD}},{\rm{LW}},0}* {\alpha }_{{\rm{LW}},{{\rm{T}}}_{\min },0}=\frac{\,\text{dLW}}{\text{dAOD}\,}* \frac{{{\rm{dT}}}_{\min }}{\,\text{dLW}\,}\end{array}$$

Although estimated for a 150 year equilibrium, we use the equilibrium climate sensitivity (ECS) to approximate the magnitude of $\frac{{{\rm{dT}}}_{\min }}{\,\text{dLW}\,}$. ECS describes the equilibrium global average temperature change for a doubling (usually relative to pre-industrial, i.e., 1850-1900) of the atmospheric CO_2_ concentration, which corresponds to a LW forcing of about 3.7 Wm^−2^ ^78^. Current estimates of ECS range between 1.5 and 4.5 K. Estimates for ${(\frac{\text{dLW}}{\text{dAOD}})}^{2014-1850}$, which is the radiative effect of ARI on LW, are taken from^19^: 0.13 ± 0.06 Wm^−2^. Since ARI_LW_ is calculated for an AOD change of 2014 relative to 1850, which is approximately 0.05 change of one unit AOD (derived from daily CMIP6 simulations of AOD for CanESM5 and HadGEM3), we have to multiply this value by 20 to approximate *α*_AOD,LW,0_, which describes the response in LW to a change of one unit AOD. Thus, ${\alpha }_{{\rm{AOD}}},{{\rm{T}}}_{\min },0$ is estimated to be (by order of magnitude):

11 $$\alpha_{ {\rm{AOD}},{\rm{T}}_{\min},0} = \underbrace{ {\rm{ARI}}_{{\rm{LW}}} * 20 }_{\alpha_{{\rm{AOD}},{\rm{LW}},0}} * \underbrace{ {\rm{ECS}}/{3.7} }_{ \alpha_{ {\rm{LW}},{\rm{T}}_{{{\min}}},0} } = [0.4;4.6] {\rm{K}}/{\rm{AOD}}$$

## Supplementary information

Supplemental Material

## Data Availability

All datasets analysed during the current study are freely available online: Observational data from the Climate Research Unit’s gridded Time Series v4.05 (CRUTSv4.05,^79^) are freely available online (see https://catalogue.ceda.ac.uk/uuid/c26a65020a5e4b80b20018f148556681). CMIP6^36^ model data is freely available through the Earth System Grid (see https://esgf-node.llnl.gov/projects/cmip6/). MACv2^60^ is available at ftp://ftp-projects.mpimet.mpg.de/aerocom/climatology/MACv2_2018/. CERES Syn1deg^46,47^ data can be downloaded from https://ceres.larc.nasa.gov/data/. ECA&D station data^42,43^ is freely available at https://climexp.knmi.nl/selectdailyseries.cgi?id=someone@somewhereand E-OBS^56^ at https://www.ecad.eu/dailydata/index.php. CAMS global reanalysis of AOD, sulfate and black carbon can be downloaded from the Copernicus Atmosphere Data Store (https://ads.atmosphere.copernicus.eu/datasets/cams-global-reanalysis-eac4?tab=overview).

## Code availability

The underlying code and processed data for this study are available on GitHub and can be accessed via this link https://github.com/cmroesch/DTR-causal-analysis.
