# Supplementary material for: Decreasing aerosols increase the European summer diurnal temperature range
Source: NPJ Clim Atmos Sci. 2025 Feb 12;8(1):47. doi: 10.1038/s41612-025-00922-3 (PMC11821512; doi:10.1038/s41612-025-00922-3)
Supplement: Supplementary file 1 — Supplemental Material [file 41612_2025_922_MOESM1_ESM.pdf]

Supplementary material

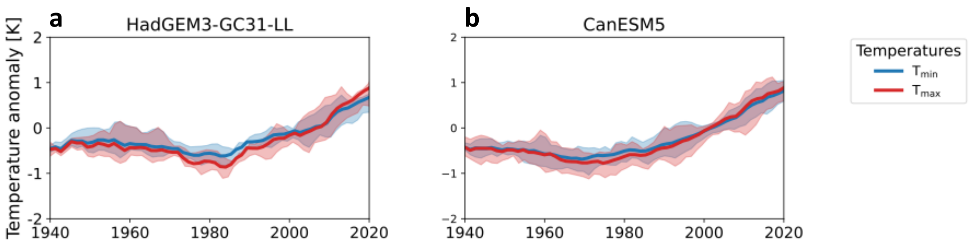

Fig. S1 Anomalies of daily maximum (red; T_{max}) and minimum (blue; T_{min}) temperature for **a** HadGEM3-GC31-LL and **b** CanESM5.

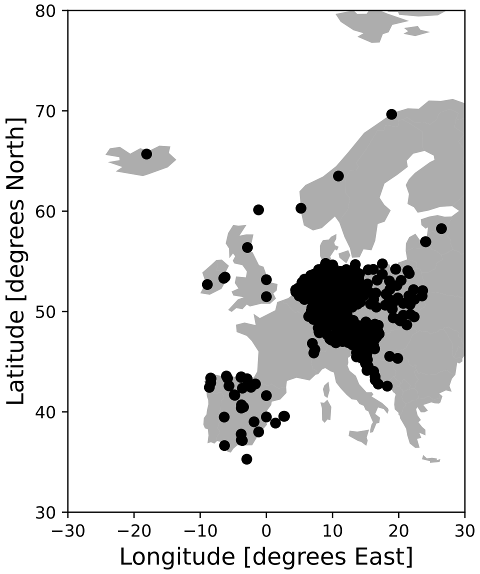

Fig. S2 Locations of the ECA&D stations [49, 50]. Stations are filtered for having at least 10 years of available MJJA daily data between 2001 and 2021.

Table S1 Link assumptions for the graphs in Supplementary Figure S6. Link assumptions are set based on our physical understanding of aerosol effects in the climate system.

Graph	Link assumptions
a	none
b	no direct link from AOD to T_{max}, AOD can only be impacted by clouds, clouds can only be impacted by AOD, SW can only be impacted by AOD and clouds, no direct link between SW and T_{min} as T_{min} is measured before sunrise, T_{min} and T_{max} can impact each other but no other variable
c	same as in b and T_{max} drives T_{min} at lag 1

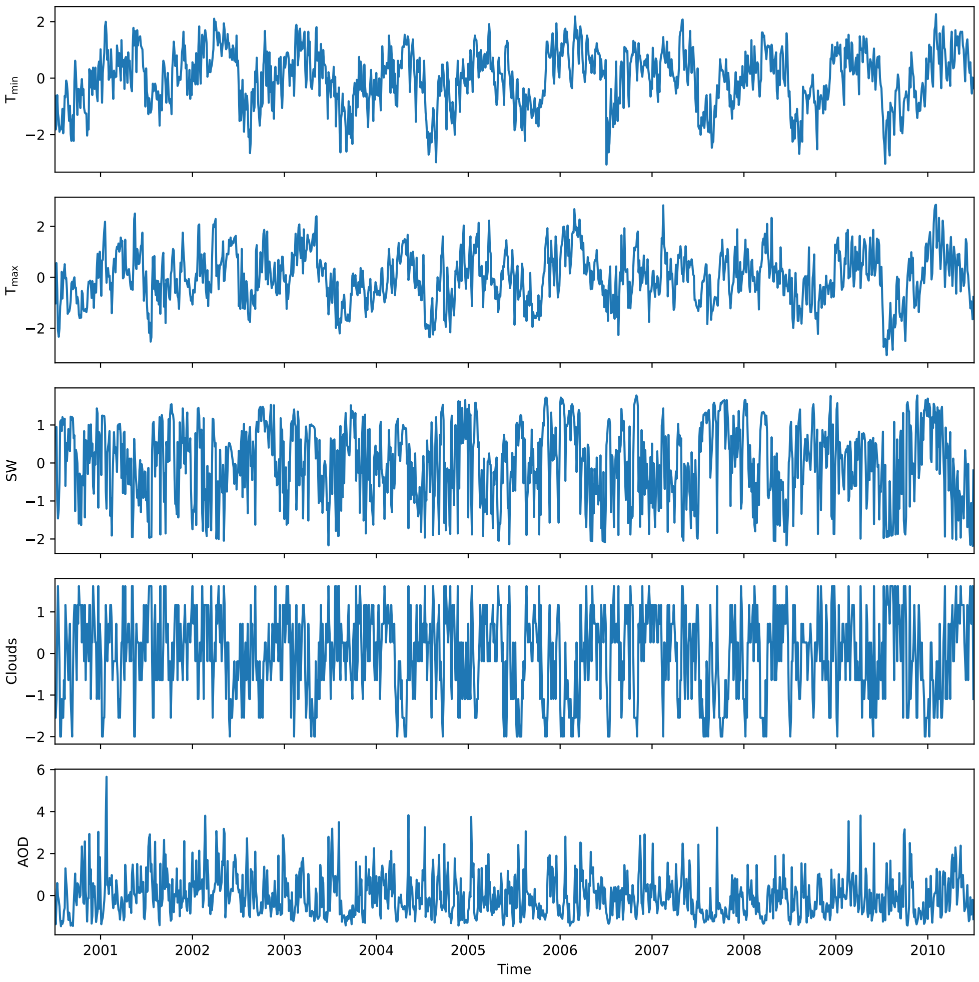

Fig. S3 MJJA timeseries of T_{min}, T_{max}, SW, Clouds and AOD for the ECA&D station (id = 266) in Bodo (Norway) for 2001-2010. Timeseries are formed by concatenating standardized (w.r.t. station and year) MJJA timeseries of individual years.

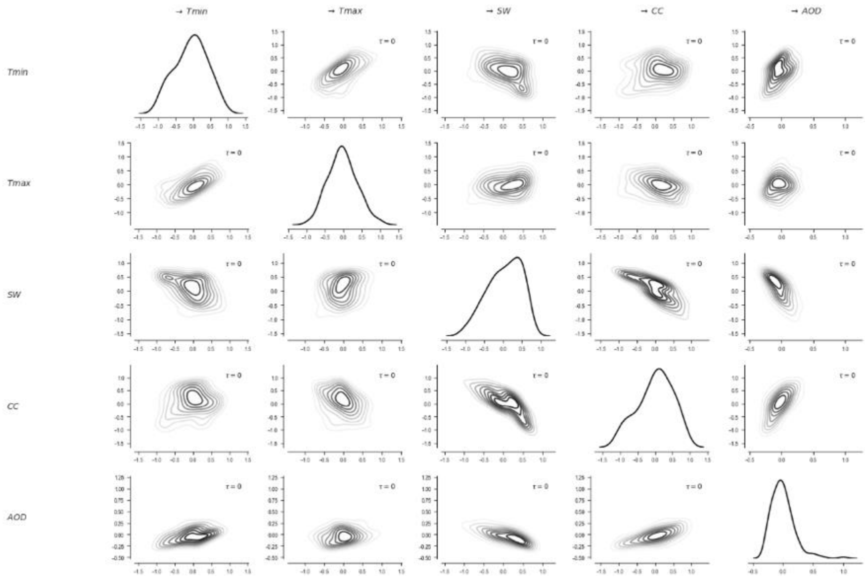

Fig. S4 Density plots of correlations between variables. The correlation and autocorrelation (diagonal) between the different causal nodes are displayed. The data are standardized before the correlation analysis.

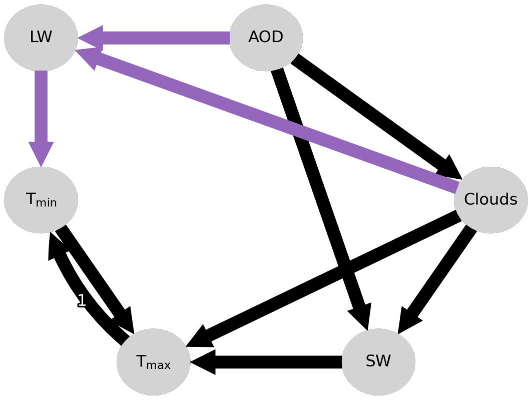

Fig. S5 Theoretical causal graph including longwave radiation (LW; purple). The graph is derived based on the discovered causal graph from Figure 2a and expert knowledge on the effects of LW radiation. Arrows represent causal links and circles causal nodes. Curved arrows depict lagged links with the lag (in days) provided as a number. Black links are discovered during the causal discovery. Purple links are theoretical and cannot be resolved due to data limitations, and are hence implicitly included in links 6 and 7 in Figure 2a.

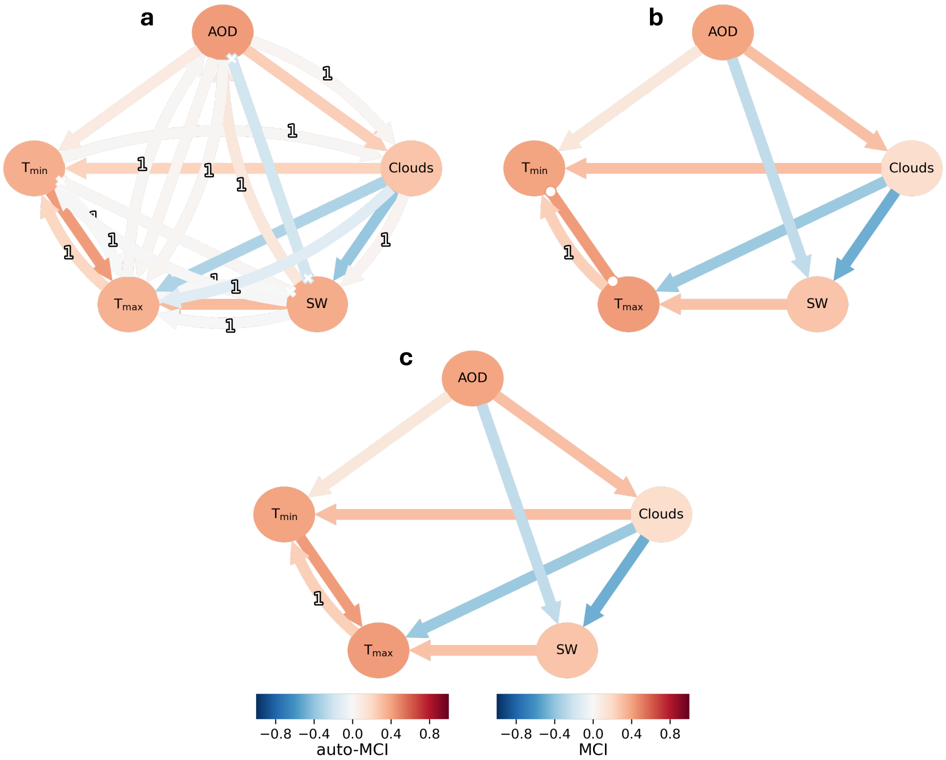

Fig. S6 Comparison of discovered causal graphs for different levels of link assumptions. Link assumptions are imposed during causal discovery to prevent spurious links and improve the performance of the PCMCI+ algorithm. In **a** no assumptions are made while **b** shows a discovered causal graph where physical constraints (listed in Supplementary Table S1) are imposed. The graph in **c** has the same constraints as b but T_{min} and T_{max} are also ordered in time. Circles depict causal nodes and the direction of the arrows shows the direction of the causal effects. The color of the arrows depicts the correlation sign (MCI), color of the nodes the autocorrelation from the previous time step (auto-MCI) and curved arrows highlight lagged links with the time lag marked as an outlined number. Note that reduced links are weak if included, confirming our graph.

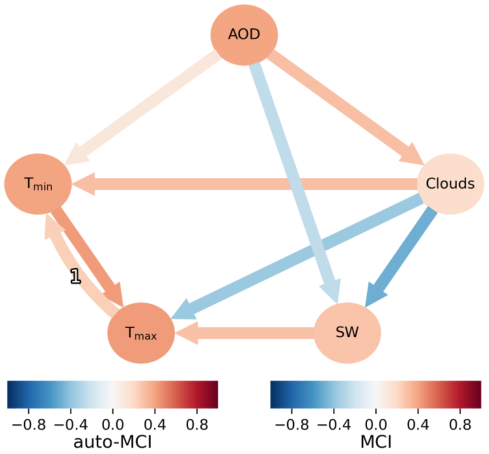

Fig. S7 Discovered causal graph. Same as Figure 3a but for Boreal summer season (April to October) instead of summer (MJJA). The color of the arrows depicts the correlation sign and strength (MCI), color of the nodes the autocorrelation from the previous time step (auto-MCI) and curved arrows highlight lagged links with the time lag marked as an outlined number. Note that reduced links are weak if included, confirming our graph.

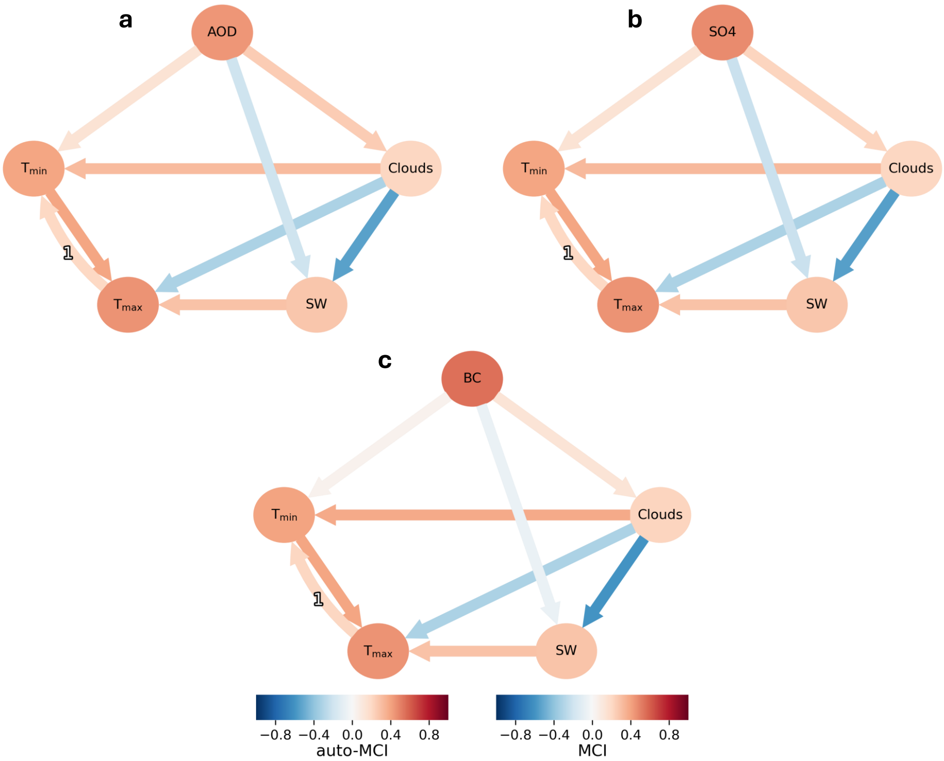

Fig. S8 Comparison of discovered causal graphs for **a** AOD, **b** sulfate (SO_4) and **c** black carbon (BC). The color of the arrows depicts the correlation sign (MCI), color of the nodes the autocorrelation from the previous time step (auto-MCI) and curved arrows highlight lagged links with the time lag marked as an outlined number. Note that reduced links are weak if included, confirming our graph.

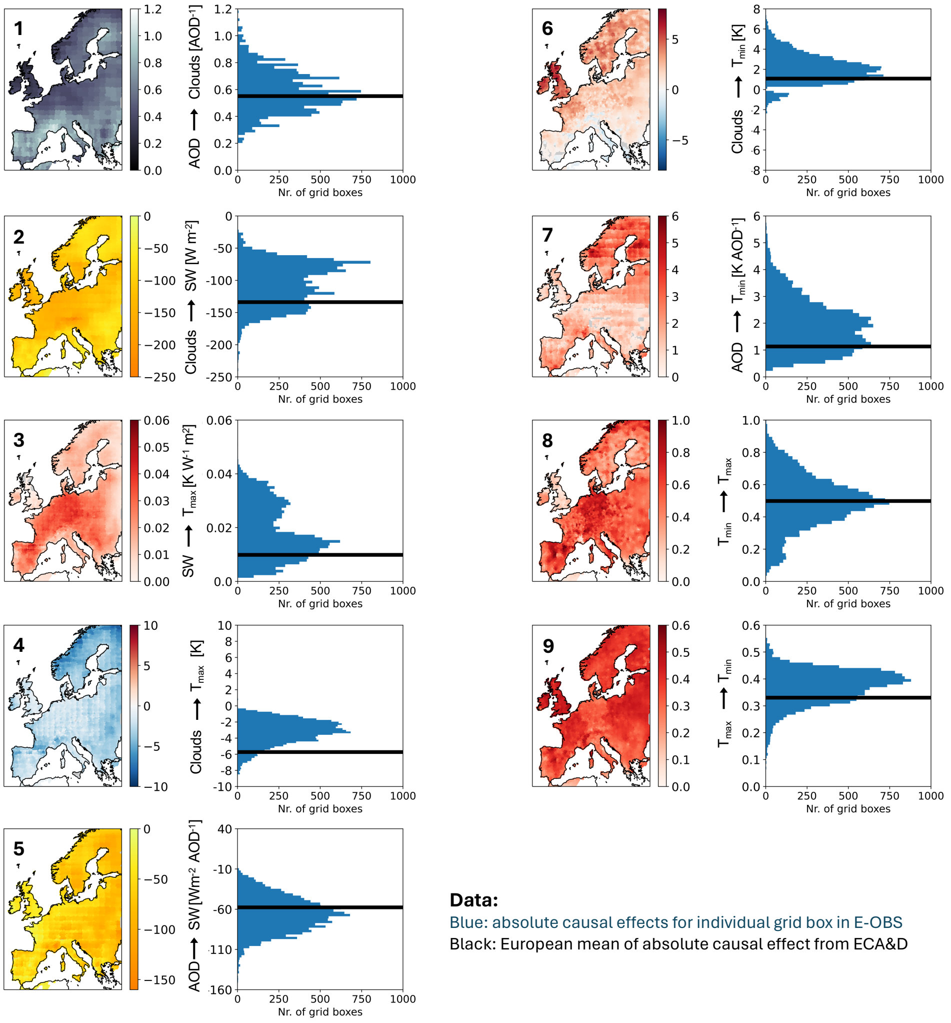

Fig. S9 Estimated causal effects for Europe using E-OBSv27.0e. Maps show heatmaps of the causal effects, histograms the distributions of the causal effects across Europe. Black lines show the mean European causal effect estimated using ECA&D station data. Numbers refer to the causal links in Figure 2a and Table 1. Note that the strong deviation of the European mean (ECA&D station data; black line) from the peak of the distribution in 4, is due to the different units of the cloud dataset in E-OBS (cloud area fraction) and ECA&D station data (Okta/8).

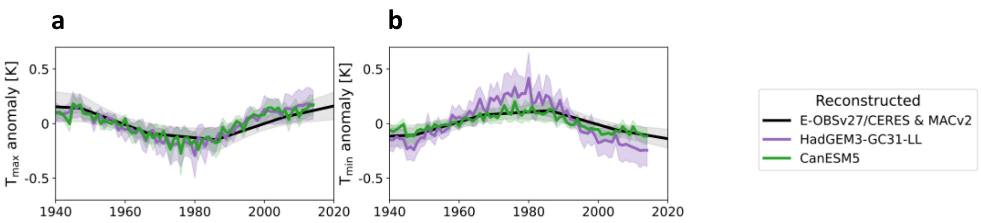

Fig. S10 Estimated aerosol causal effects. In **a** aerosol effects on T_{max} (1940-2014 for CMIP6 models in blue and purple; 1940-2020 for E-OBS and MAC; solid lines) and **b** on T_{min}. Total causal effects of aerosols on T_{max} and T_{min}, which are used to estimate aerosol effects, are shown in Figure 3 **b** and **c**, respectively.
